# Cervicomedullary angle as an independent radiological predictor of postoperative neurological outcome in type A basilar invagination

**DOI:** 10.1038/s41598-019-55780-w

**Published:** 2019-12-18

**Authors:** Xiang Guo, Zhao Han, Jiajia Xiao, Qunxiang Chen, Fei Chen, Qunfeng Guo, Jun Yang, Bin Ni

**Affiliations:** 1 Department of Orthopedics, Changzheng Hospital, The Second Military Medical University, Shanghai, People’s Republic of China; 2 Department of General medicine, The Second affiliated hospital of Xi’an Jiaotong University, Shannxi, People’s Republic of China

**Keywords:** Spine plasticity, Outcomes research

## Abstract

To propose an independent radiological index to evaluate surgical outcomes of A type basilar invagination, a retrospective study was conducted to compare the clinical outcome between procedures 1 and 2 by applying intraoperative consistent traction and manual reduction. Moreover, the atlantodental interval (ADI), cervicomedullary angle (CMA), bilateral sagittal inclination of atlantoaxial joint (SIAA) were measured and compared to pre-operation. Postoperatively, only these patients undergoing procedure 2 achieved significant neurological improvement. The ADIs and the SIAAs decreased in both groups, these differences are statistically significant between pre- and post- operation. For postoperative CMAs, only these patients undergoing modified surgery gained significant improvement of angle with mean 141°. We concluded that the CMA or SIAA could be a radiological predictor to evaluate surgical outcome in BI, among which the CMA is a more independent and easily measurable predictor that is closely correlated with satisfactory neurological improvements. Moreover, procedure 2 with intraoperative resistant cranial traction and manual reduction can help us achieve a good CMA.

## Introduction

Basilar invagination (BI) is a complex occipitocervical deformity. According to Goel’s studies^1,2^, BI is composed of 2 types. Type A basilar invagination is characterized by atlantoaxial instability, accompanied by dislocation of the odontoid process into the foramen magnum according to the Chamberlain and Wackenheim lines^3^. Type B basilar invagination is characterized by basilar invagination, but there is no sign of atlantoaxial instability evaluated by an abnormal increase in the atlantodental interval (ADI).

The initial diagnosis of basilar invagination occurs via plain films. Generally, if the atlantoaxial complex cannot be seen on plain anteroposterior films, the possibility of basilar invagination is suspected. Further diagnoses rely on several lines on the cervical spine: the Chamberlain line, McGregor line, and McRae line^4–6^. The Chamberlain line is also called the occipitocervical line, which is the connection between the posterior edge of the hard palate and the posterior border of the foramen magnum. The normal odontoid tip is below the line. If the odontoid tip exceeds the occipitocervical line by more than 5 mm, a diagnosis of basilar invagination is established. Because it is difficult to determine the posterior edge of the foramen magnum on plain films, McGregor modified the Chamberlain line from the posterior edge of the hard palate to the lowest point of the squamous part of the occipital bone if the odontoid tip moves upward beyond this line by 7 mm, which is abnormal. The McRae line is the line connecting the anterior and posterior borders of the foramen magnum; normally, the odontoid process does not exceed this line.

Basilar invagination normally results in serious compression of the spinal cord, and surgical intervention is required immediately after diagnosis. The aim of surgery is to reduce dislocation of atlantoaxial articulations, relieving spinal cord compression and rebuilding instability of the occipitocervical region. However, after surgical intervention, some patients can achieve significant neurological improvements, while others cannot. This phenomenon makes it imperative for us to continually modify the surgical techniques and explore what factors are correlated with clinical outcome. Several previous studies^7–12^ have shown that patients with BI have deformities of odontoid or/and lateral mass joint,and proposed several parameters to assess deformity morphology, further investigated the correlation between deformities of the odontoid or lateral mass joints and the severity of BI. In this study, the authors not only compared the difference in clinical outcome between procedure 1 and procedure 2 but also focused on the measurement of several radiological predictors, such as the ADI, cervicomedullary angle (CMA), and sagittal inclination of the atlantoaxial joint (SIAA). Furthermore, this study investigated the correlation between these variations of anatomic parameters and surgical outcomes to screen out a reliable evaluation predictor for the treatment of type A basilar invagination.

## Methods and Materials

The Ethics Committee of our hospital approved this study, and signed informed consent was provided by all patients for their information to be stored in the hospital database and used for research. We confirm that all methods were performed in accordance with the relevant guidelines and regulations.

A retrospective study was conducted to compare differences in clinical outcomes between procedures 1 and 2 by applying intraoperative consistent traction and manual reduction. The correlation between variations of anatomic parameters and clinical outcomes was also evaluated. In a series of cases, 21 patients who were diagnosed as basilar invagination were admitted. The ages was from 25 to 65 years, the average age was 43. The inclusion criteria were ① atlantoaxial instability, ② without bone fusion of atlantoaxial articulations ③ without a history of cervical surgery. Patients with cervical cord compression due to tumors, trauma, ossification of the posterior longitudinal ligament (OPLL) or infection were excluded. The series of patients was divided into two groups according to the different surgical techniques. One group included 6 patients who underwent procedure 1 (P1) without intraoperative consistent cranial traction and manual reduction; the other group, which was managed by the latest authors and involved a customized intraoperative traction appliance and a mature reduction technique that had been developed, included 15 patients who underwent procedure 2 (P2). All patients’ clinical symptoms and neurological functions were compared both at preoperative and the final point of follow-up. Cervical pain was assessed using the Visual Analogue Scale (VAS). Neurological function was assessed using the ASIA impairment scale ranking (2011). Additionally, complications related to surgery were investigated.

**Figure 1:**
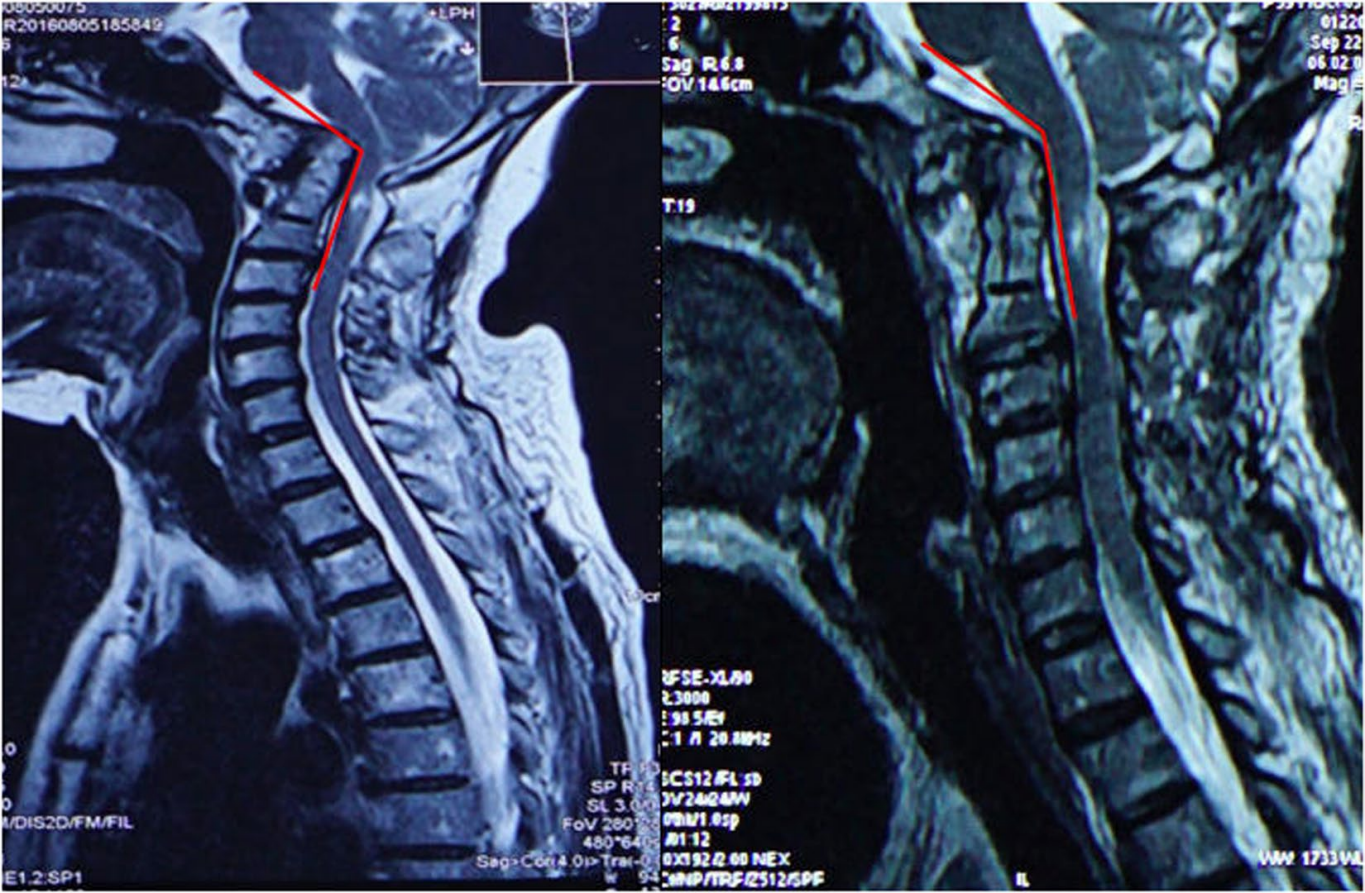
The CMA is defined as the included angle between the line parallel to ventral side of medulla oblongata and the line parallel to the ventral side of the upper cervical cord in MRI of cervical spine. An MRI sagittal imaging showed that CMA had changed pre-(left) and post-operation (right).

**Figure 2:**
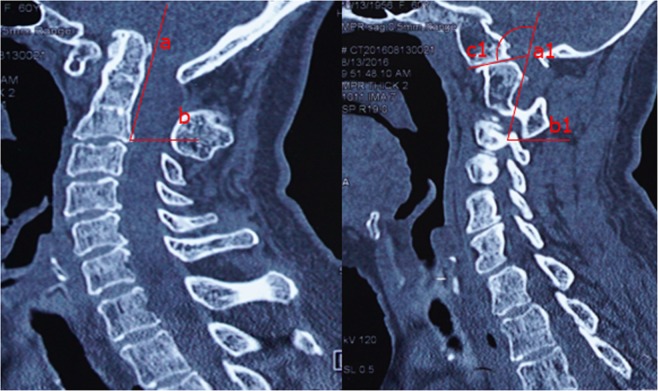
The SIAA is the angle between the long axis of the posterior edge of the odontoid process (midsagittal section) and C1/C2 joint (parasagittal section). A CT sagittal reconstruction imaging showed the measurement procedure of preoperative SIAA.

Apart from the abovementioned clinical outcomes, comprehensive radiological measurements were made. Specifically, cervical radiographs, MRI and CT scans with three-dimensional reconstructions were performed, and changes in related imaging parameters, including the CMA and SIAA (bilateral side), were evaluated both at preoperative and the final point of follow-up. The CMA is defined as the included angle between the line parallel to the ventral side of the medulla oblongata and the line parallel to the ventral side of the upper cervical cord on MRI of the cervical spine (Fig. 1)^13^. The SIAA is the angle between the long axis of the posterior edge of the odontoid process (midsagittal section) and the C1/C2 joint (parasagittal section). However, because the odontoid process and C1/C2 joint are in different sections, a special method was used to measure the sagittal inclination^7^: first, the angle subtended of the line of the posterior border of the odontoid process (a) and horizontal line (b) was measured in the midsagittal section. Then, in the parasagittal section (where the joint was visible), a line (b1) was first drawn parallel to the horizontal line. Next, the same angle value was constructed as the angle in the midsagittal section. Finally, a line (c1) was drawn that passed parallel to the C2 facet joint (Fig. 2). Moreover, bone fusion was determined on the basis of the presence of trabeculated bone in the sagittally reconstructed CT images.

SPSS Statistics for Windows, Version 23.0 (SPSS Inc., Chicago, Illinois) was used to analyze the data as the mean ± standard deviation (mean ± SD). A one-way analysis of variance (ANOVA) was used to compare multiple data sets, and a paired t-test was used to perform pairwise comparisons. Differences were considered significant if the *P* value was smaller than 0.05.

### Preoperative preparation and surgical procedure

Preoperative cranial traction was required to reduce atlantoaxial dislocation in both the sagittal plane (anterior dislocation) and the axial plane (vertical dislocation). Normally, the effective traction strength is approximately 1/8–1/10 of the patient’s body weight, and the traction duration is less than two weeks. At the end of the traction period, cervical anteroposterior and lateral dynamic radiography was conducted to evaluate the reduction of the atlantoaxial joint. In our study, procedure 1, which merely involved preoperative cranial traction without intraoperative consistent cranial traction and manual reduction, was implemented in 6 patients at an earlier time. The other 15 patients underwent procedure 2 at the latest time, in which consistent cranial traction were used both in preoperative and in intraoperative period and intraoperative manual reduction was conducted. The details of this procedure is described below.

The patient was placed in the prone position while cranial traction was maintained by facilitating intraoperative traction in order to maintain C1–C2 reduction. A posterior midline cut was made from the C0 to the C2 spinal process. According to the standard technique^14–16^, C2 isthmic screws and an occipital plate (DePuy Synthes, USA) were placed under monitoring with C-arm fluoroscopy or radiography. Then, a part of the posterior arch of C1 (less than 1 cm lateral to the median bilaterally) was resected carefully to achieve decompression of the spinal canal. The precurved (approximately 90 degrees) rod was cut to the appropriate size, fitted into the open top of the C1–2 screws and twisted tightly. Then, a drop was formed between the open top of the occipital plate and the precurved rods, and the drop was used a lever to further reduce anterior dislocation of the atlantoaxial joint by manual extension of the skull when the occipital plate nuts were twisted tightly.

Finally, we removed the cortical bone at the occipital scale and C2 spinous process, an iliac bone block was harvested and shaped. The bone block was wedged between the occipital scale and the C2 spinous process and was fixed to the occipital bone with a screw.

The time of surgery ranged from 70 to 90 minutes. Blood loss was approximately 400 ml. All patients required Philadelphia collar support for three months.

## Results

The patients’ general information is summarized in Table 1. At the final follow-up, bone fusion was obtained in all patients. All patients showed pain relief and neurological improvement. There were no complications related to the operation. No patient required reoperation.

**Table 1:** Patients’ general information.

Information	Procedure 1 group (P1)	Procedure 2 group (P2)
No. of patients	6	15
Sex (male/female)	4/2	10/5
Age at operation (years)	38.17 ± 16.03	46.33 ± 11.91
Follow-up time (months)	24.3 ± 2.2	26.4 ± 4.1
Operation time (mins)	95 ± 12.5	105 ± 17*
Blood loss (ml)*	440 ± 20.8	380 ± 40.6*

Data expressed as mean ± standard deviation unless otherwise indicated.

*P < 0.05, comparison between groups using Student’s t test.

### Clinical outcomes

All patients achieved a minimum two-year follow-up. The results are summarized in Table 2.

**Table 2:** Functional outcomes of two groups.

Variable	Procedure 1 group (P1)		Procedure 2 group (P2)	
	Preoperative	Postoperative	Preoperative	Postoperative
VAS (average)	4.83	0.6*	4.8	0.4*
ASIA Scale	D	D	D	E
ASIA score sensation (light touch)	109.33 ± 1.84	109.66 ± 0.51	108.47 ± 2.13	111.66 ± 0.9*
ASIA score sensation (pin prick)	108.45 ± 1.45	108.5 ± 0.78	107.34 ± 2.03	112.23 ± 2.03 *
ASIA score motion	93.33 ± 1.36	94.46 ± 1.99	93.13 ± 2.26	98.8 ± 1.27*
ADI (MM)	6.5 ± 0.63	4.33 ± 0.26*	6.23 ± 0.61	3.92 ± 0.59*
CMA	124 ± 1.86	131 ± 0.98	125 ± 6.57	141 ± 2.63*
Left SIAA	111 ± 3.76	98.33 ± 2.25*	110.5 ± 3.18*	86.3 ± 5.3*
Right SIAA	109 ± 4.51	96.16 ± 5.52*	109 ± 3.18*	85.87 ± 3.48*

Data expressed as mean ± standard deviation unless otherwise indicated.

ADI: Atlas-Dens Interval.

CMA: Cervicomedullary angle.

SIAA: Sagittal Inclination of Atlantoaxial Joint.

*Represent *P* < 0.05.

Before surgery, all patients complained of suboccipital pain or neck pain accompanied by different degrees of myelopathy. After surgery, up to 95% of patients with neck and suboccipital pain demonstrated improvements in their VAS pain scores, with a greater than 50% improvement in their VAS scores and a decrease of 5 points on the VAS. There was a statistically significant difference between their pre- and postoperative pain scores (P < 0.05).

The preoperative ASIA impairment scale of these 21 patients was D. After surgery, there was an improvement in the majority of the patient’s myelopathy grade: 15 patients were grade E, and 6 patients were grade D (unchanged). For procedure 1, the preoperative mean motor score of 93.33, light touch score of 109.33, and pin prick score of 108.45 were slightly improved, with a postoperative mean motor score of 94.46, light touch score of 109.66 and pin prick score of 108.5; the difference was not statistically significant (P > 0.05). For procedure 2, the preoperative mean motor score of 93.13, light touch score of 108.47, and pin prick score of 107.34 were significantly improved, with a postoperative mean motor score of 98.8, light touch score of 111.66, and pin prick score of 112.23; the difference was statistically significant (P < 0.05).

No implant failure or neurovascular injury occurred. Although there was no mortality found in this series preoperatively, at the time of follow-up, 2 patients were found to have died, but their deaths were unrelated to their surgery and occurred more than 6 months after surgery.

### Radiological outcomes

**Figure 3:**
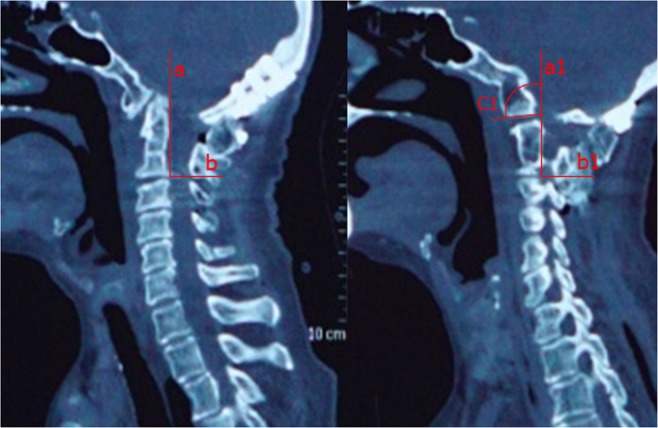
A CT sagittal reconstruction imaging showing the measurement procedure of postoperative SIAA. The arrow marked the bone graft.

The ADI decreased in all patients. For the procedure 1 group, the preoperative mean was 6.5 mm compared with the postoperative mean of 4.33 mm, in which there were 2 patients (23%) with instability appearance in the atlantoaxial joint on the lateral flexion-extension radiograph at follow-up (ADI >4.0 mm); for the procedure 2 group, the preoperative mean was 6.23 mm compared with the postoperative mean of 3.92 mm (ADI >4.0 mm). The CMA increased in all patients. For the procedure 1 surgery group, the preoperative mean angle was 124° compared with the mean postoperative angle of 131°; the difference was not statistically significant. Three patients (9.5%) still had signal changes within the spinal cord on T2WI MRI six months after surgery. For the procedure 2 group, the preoperative mean angle was 125° compared with the postoperative mean angle of 141°; the difference was statistically significant. The bilateral sagittal inclination of the atlantoaxial joint decreased in all patients (Fig. 3). For the procedure 1 group, the left preoperative mean angle was 111° compared with the postoperative mean angle of 98.33°, and the right preoperative mean angle was 109° compared with the postoperative mean angle of 96.16. For the procedure 2 group, the left preoperative mean angle was 110.5° compared with the postoperative mean angle of 86.3°, and the right preoperative mean angle was 109° compared with the postoperative mean angle of 85.87°. These differences were statistically significant (P < 0.05).

Moreover, 6 months after surgery, 20 patients (95.2%) achieved complete fusion of the bone graft in addition to 1 patient (4.8%) who suffered from osteoporosis. After being reinforced by treatment for osteoporosis and continually fixed with a collar for more than three months, the bone graft was fused.

## Discussion

With the analysis of the data of clinical outcomes and radiological measurements, an interesting finding gradually emerged. Fifteen (71.4%) patients gained significant improvements in clinical or radiological outcomes; however, 6 (28.6%) patients maintained the same status in terms of the scale or scores of preoperative neurological tests, although they also achieved improvement to some extent of chief complaints and several radiological parameters. After carefully reviewing the radiological data of these 6 patients, the authors found that although their ADIs decreased to some extent, which implied that the atlantoaxial sagittal anterior dislocation had been partly corrected, their CMAs had not changed to a reasonable level that would continue to result in compression of the spinal cord^7,17^.

What radiological parameters can affect the clinical outcomes of basilar invagination? The consensus resulting from a series of radiological studies^2,8,9,18–25^ is that the severity of BI is correlated with the extent of atlantoaxial dislocation and compression of the spinal cord. In Xia *et al*.’s study^7^, the authors proposed that deformities of the odontoid process and the lateral joint were correlated with BI severity. In particular, the SIAA was positively correlated with the severity of BI and atlantoaxial dislocation^11,12^. Hence, except for the ADI parameter, the SIAA acts as a diagnostic index and predictive index of the prognosis of BI. However, the ADI or SIAA merely describes the sagittal instability of the atlantoaxial joint but cannot explicitly manifest the extent of vertical dislocation of the atlantoaxial joint coexisting with sagittal dislocation, which is even a main cause of compression of the spinal cord. Also Bundschuh *et al*.^17^ suggested that the CMA less than 135° may result in medulla oblongata compression, cervical myelopathy, or C2 nerve root paralysis and other neurological disfunctions. Recently, Wang *et al*.^13^ proposed that CMA values obtained from midsagittal T1 MRI represented a highly reliable and repeatable measurement. Based on these studies and our clinical practices, we propose that the CMA should be considered an important diagnostic predictor of the surgical prognosis of BI that is not correlated with the ADI or SIAA. In the current study, although the 6 patients without significant neurological function improvement achieved improvement to some extent of chief complaints, their CMAs and SIAAs did not improve to a reliable level (CMA >135°); consequently, compression of the spinal cord still existed. However, the other 15 patients (72.4%) with significant neurological function improvement gained significant advances in the CMA, which implied that a satisfying reduction was present in the sagittal and axial planes. Therefore, compared to the SIAA or ADI, the CMA can more directly show the severity of spinal cord compression and is more strongly correlated with clinical outcome. Therefore, the CMA may be an independent radiological parameter, whether in making a preoperative diagnosis or evaluating postoperative clinical outcomes.

**Figure 4:**
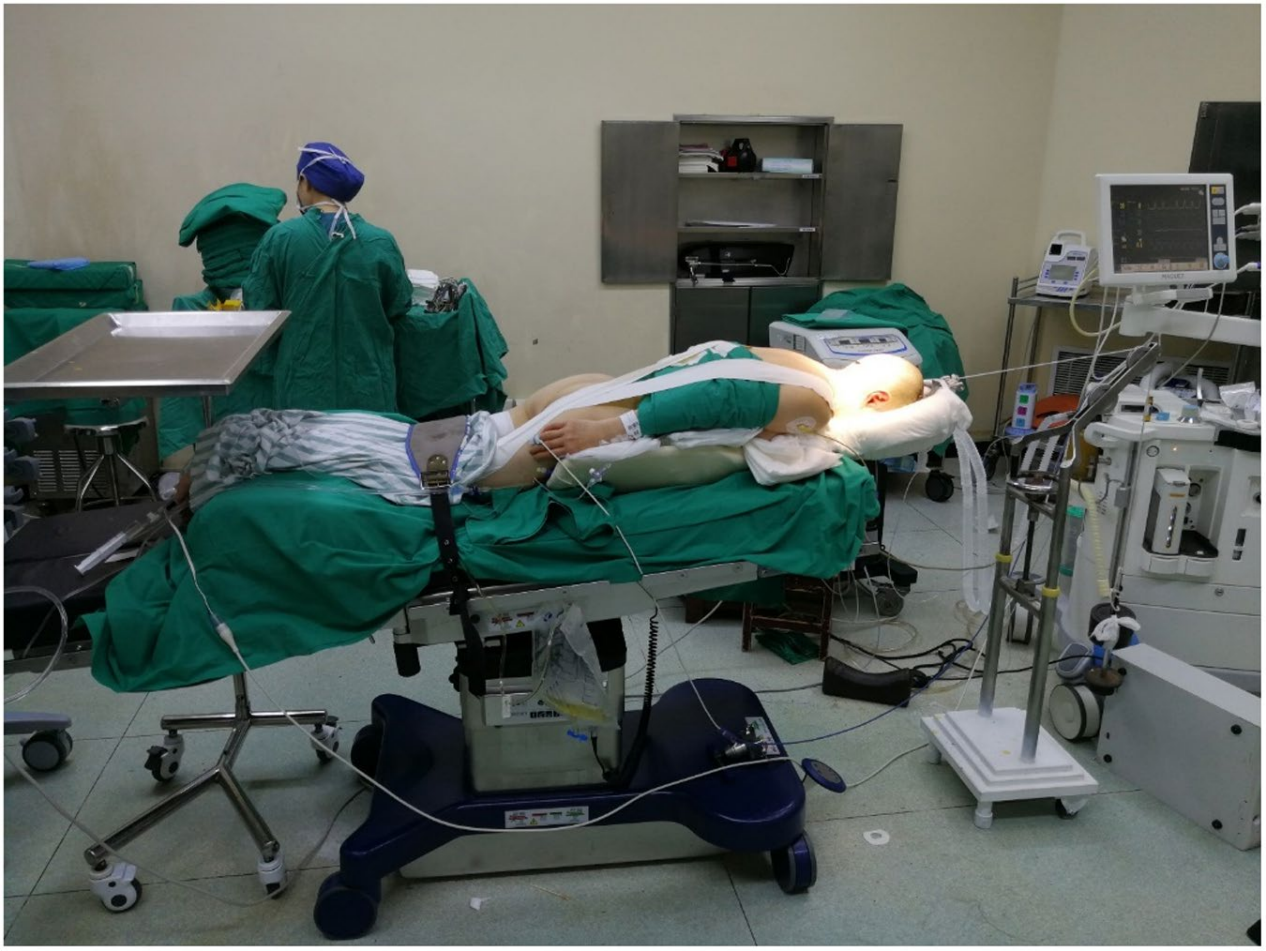
A photograph showing intraoperative resistant cranial traction after anesthesia to bring on a further reduction of atlantoaxial dislocation.

For BI with atlantoaxial instability, to obtain a satisfactory SIAA and CMA, surgical intervention is required to reduce the dislocation of the atlantoaxial joint, followed by relieving compression of the spinal cord and reconstructing spinal stability with implants. Reduction always plays an important role throughout clinical treatment. Preoperative cranial traction is an easy and effective reduction method for atlantoaxial dislocation. In the current cases, the effective traction strength was approximately 1/8–1/10 of the patient’s body weight, and the traction duration was not less than two weeks. At the end of traction, cervical anteroposterior and lateral dynamic radiography were conducted to evaluate the reduction in the atlantoaxial joint. After effective cranial traction, patients achieved reduction to varying degrees both in the sagittal and axial planes. Procedure 2 consisted of intraoperative resistant traction after anesthesia, and manual reduction operation should be used for the further reduction of atlantoaxial dislocation (Fig. 4). This intraoperative reduction technique is different from other established surgical techniques for BI with atlantoaxial dislocation^12^. Consistent intraoperative cranial traction using a customized appliance can guarantee effective reduction both in the sagittal and axial planes during surgery, which could especially result in a satisfying reduction of vertical dislocation of the atlantoaxial joint that is important for improving the CMA. In the P2 group, all patients underwent occipitocervical fusion following resection of part of the C1 posterior arch, which further enlarged the volume of the spinal canal through indirect relief of anterior compression on the spinal cord. The clinical outcome showed that 15 patients gained satisfactory improvements in the CMA and consequently had significant neurological function improvement; however, 6 patients had hardly achieved significant neurological improvement accompanied by poor improvements in the CMA. Hence, the comparative result also suggests that the CMA is a curial evaluation predictor that is closely associated with clinical outcomes, and intraoperatively consistent cranial traction can help us achieve a satisfactory improvement in the CMA. Certainly, for BI with irreducible atlantoaxial dislocation, most patients still have an unfinished reduction in the sagittal or axial plane resulting from ligament or bony hyperplasia around the atlantoaxial joint via intraoperative cranial traction and manual reduction manipulation. Therefore, the relief of anterior tissues was conducted first in or around the atlantoaxial joint for satisfactory reduction.

## Conclusions

According to our study, the CMA or SIAA could be a radiological predictor to evaluate surgical outcome in BI, among which the CMA is a more independent and easily measurable predictor that is closely correlated with satisfactory neurological improvements. Moreover, procedure 2 with intraoperative resistant cranial traction and manual reduction can help us achieve a good CMA.
